# Supplementary material for: Cytostatic Action of Novel Histone Deacetylase Inhibitors in Androgen Receptor-Null Prostate Cancer Cells
Source: Pharmaceuticals (Basel). 2021 Jan 29;14(2):103. doi: 10.3390/ph14020103 (PMC7912319; doi:10.3390/ph14020103)
Supplement: Supplementary file 1 [file pharmaceuticals-14-00103-s001.pdf]

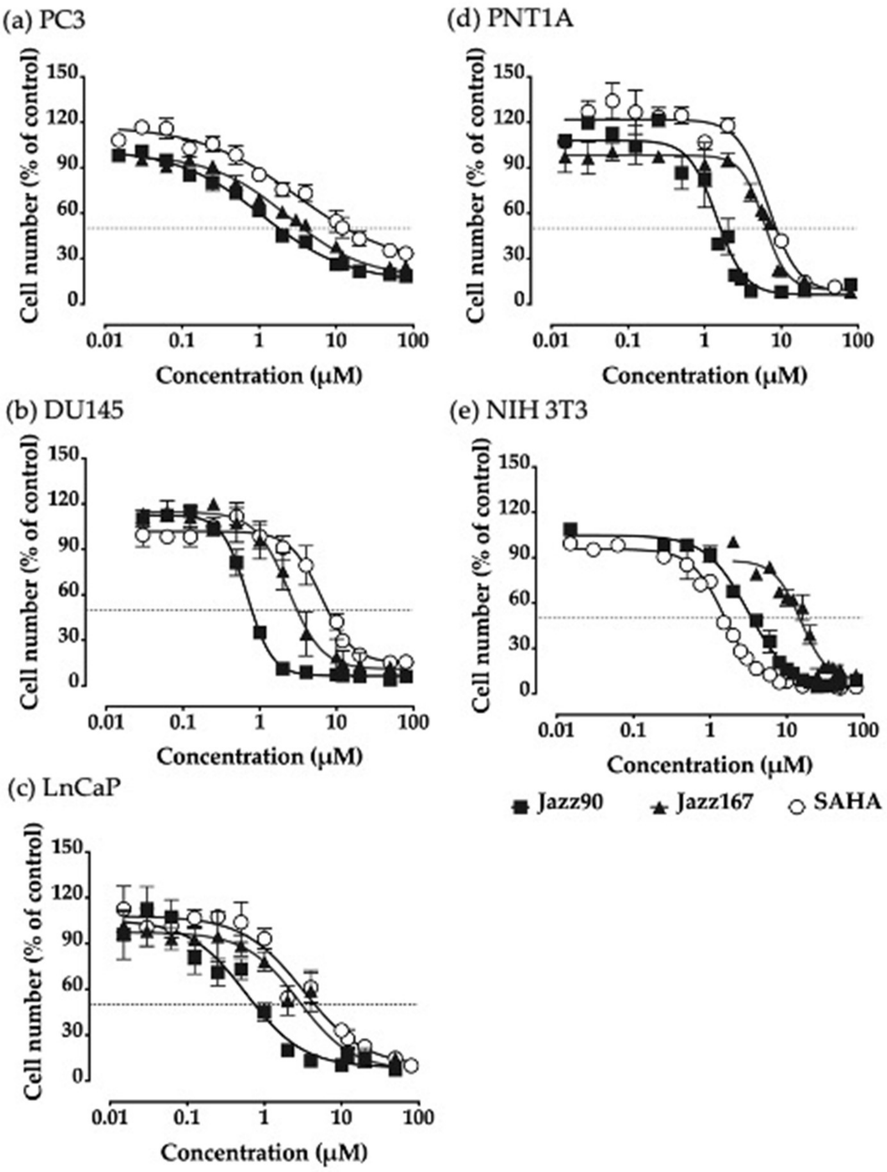

Figure S1. Cytotoxicity of Jazz90, Jazz167 and SAHA in (a) PC3, (b) DU145, (c) LnCaP, (d) PNT1A and (e) NIH 3T3 cells treated at concentrations ranging from 0–150 μM. Vehicle control cells were treated with 0.5% DMSO. After 72 hours, SRB assays were conducted. Symbols indicate cell numbers (% of control) ± S.E.M. from 3 independent experiments in triplicates. Non-linear regression was used to calculate the EC_{50} values.

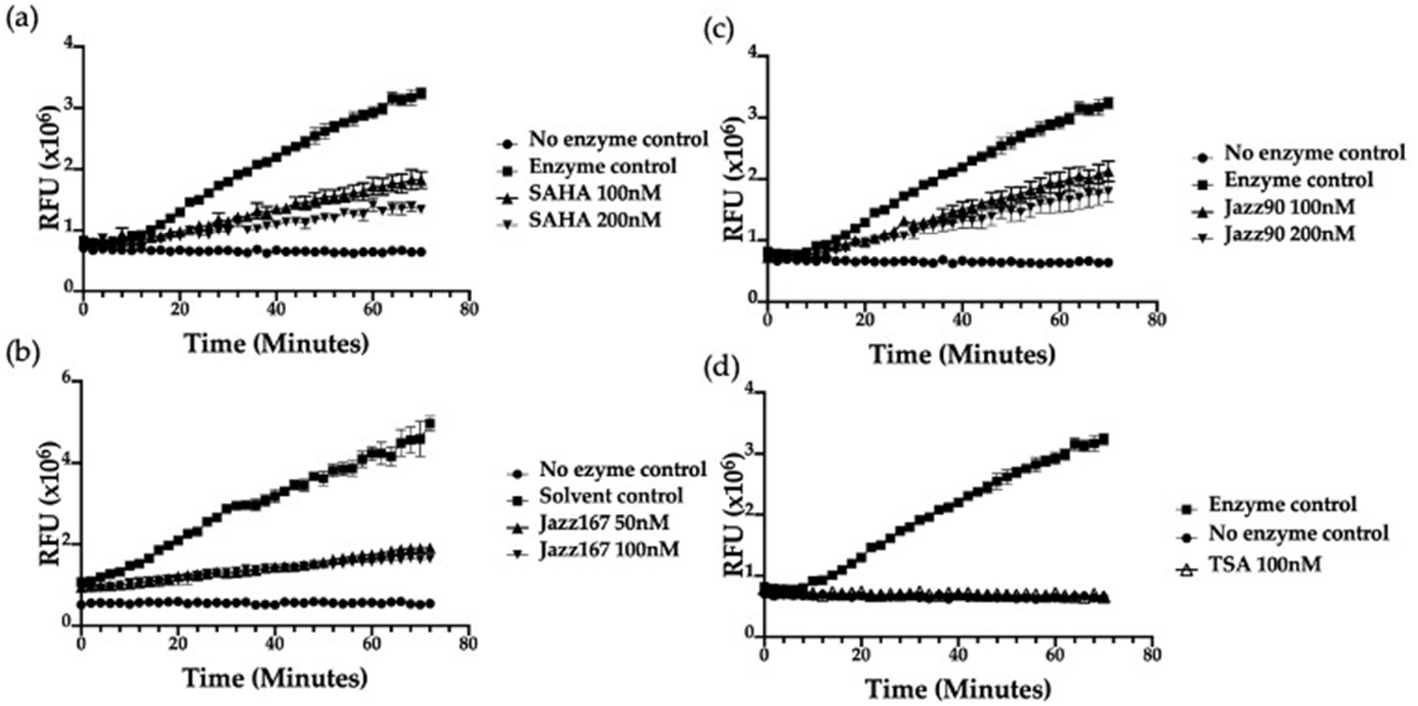

Figure S2- Kinetic assays following drug treatments (a) SAHA, (b) Jazz90, (c) Jazz167 and (d) TSA (positive control) on nuclear lysates of HeLa cells. Recordings were taken every 2 minutes for a period of 70 minutes.

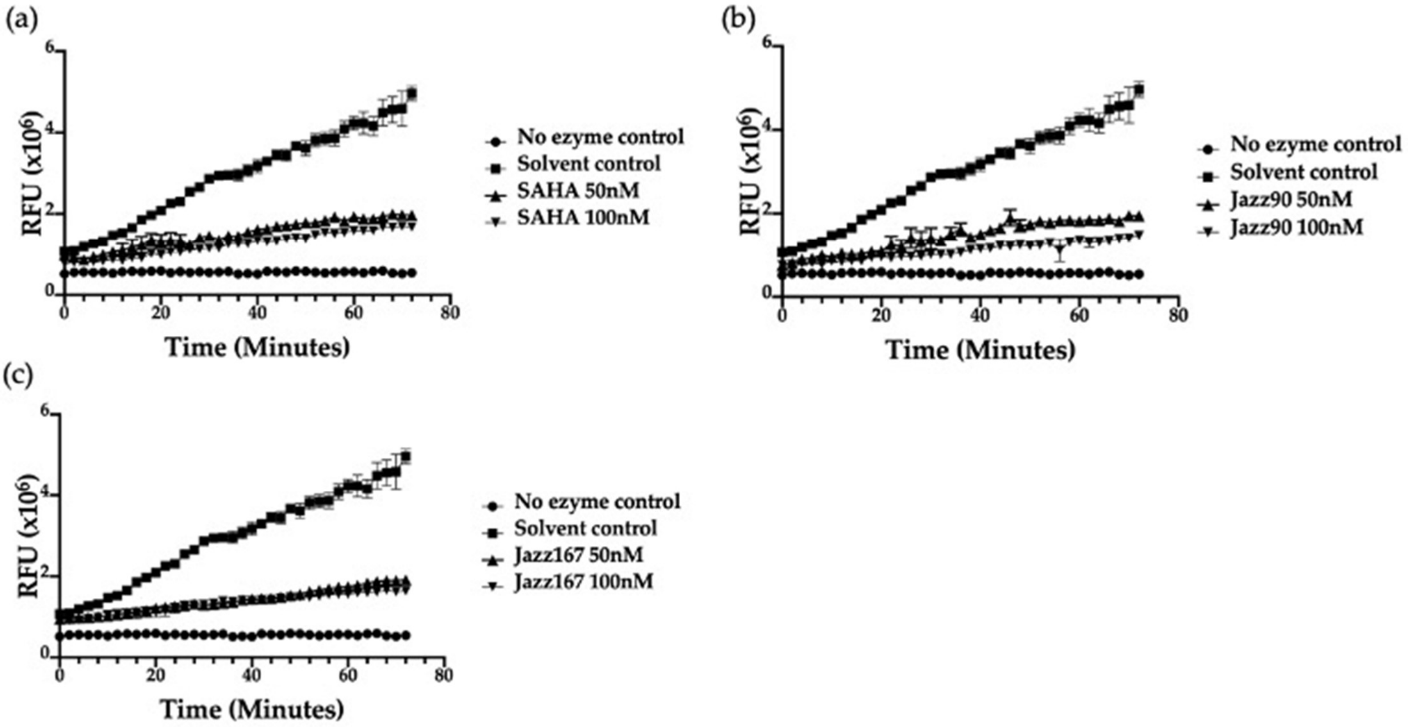

Figure S3- Kinetic assays in PC3 nuclear lysates using the drugs (a) SAHA, (b) Jazz90 and (c) Jazz167. Kinetic assays were carried out at intervals of 2 minutes for a period of 72 minutes.

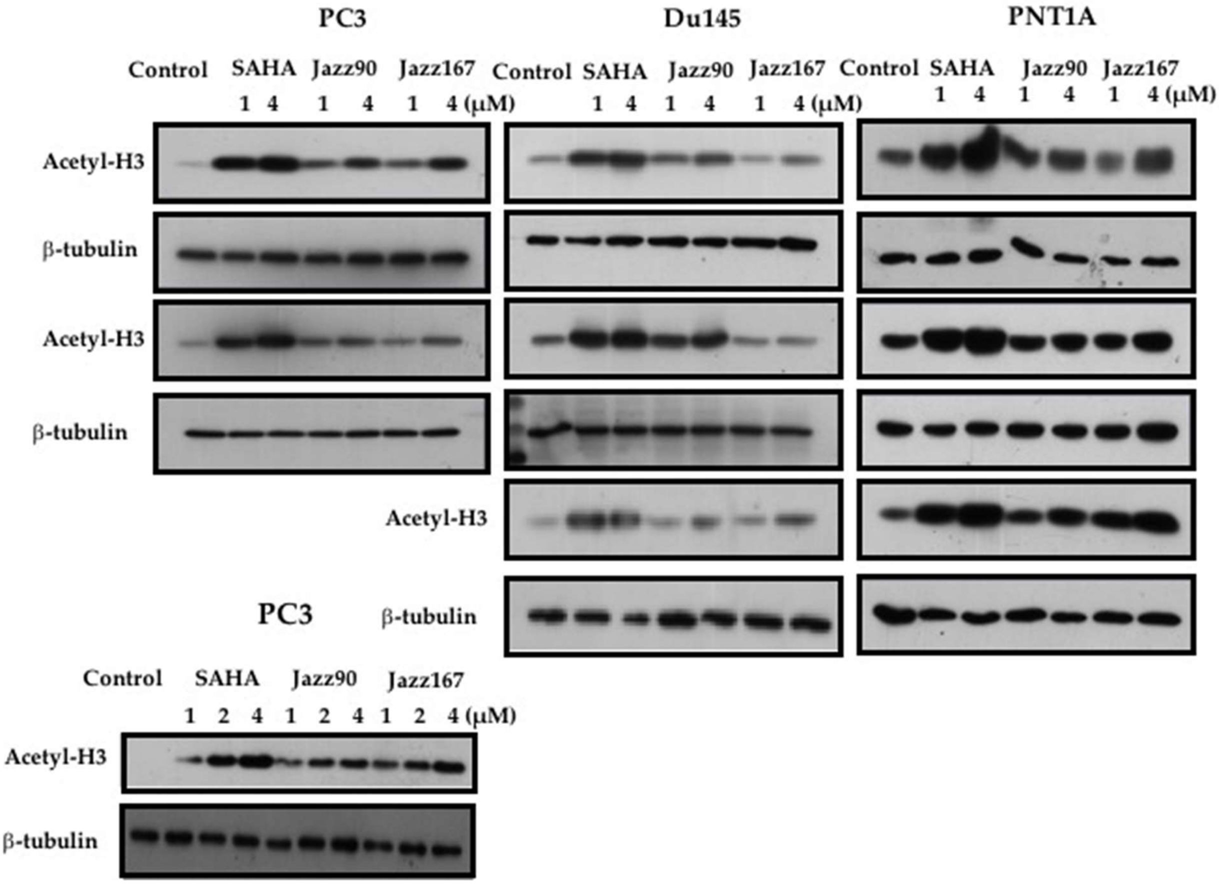

Figure S4- All blots for the acetyl-H3 protein used for densitometry. These blots were assessed via densitometry.

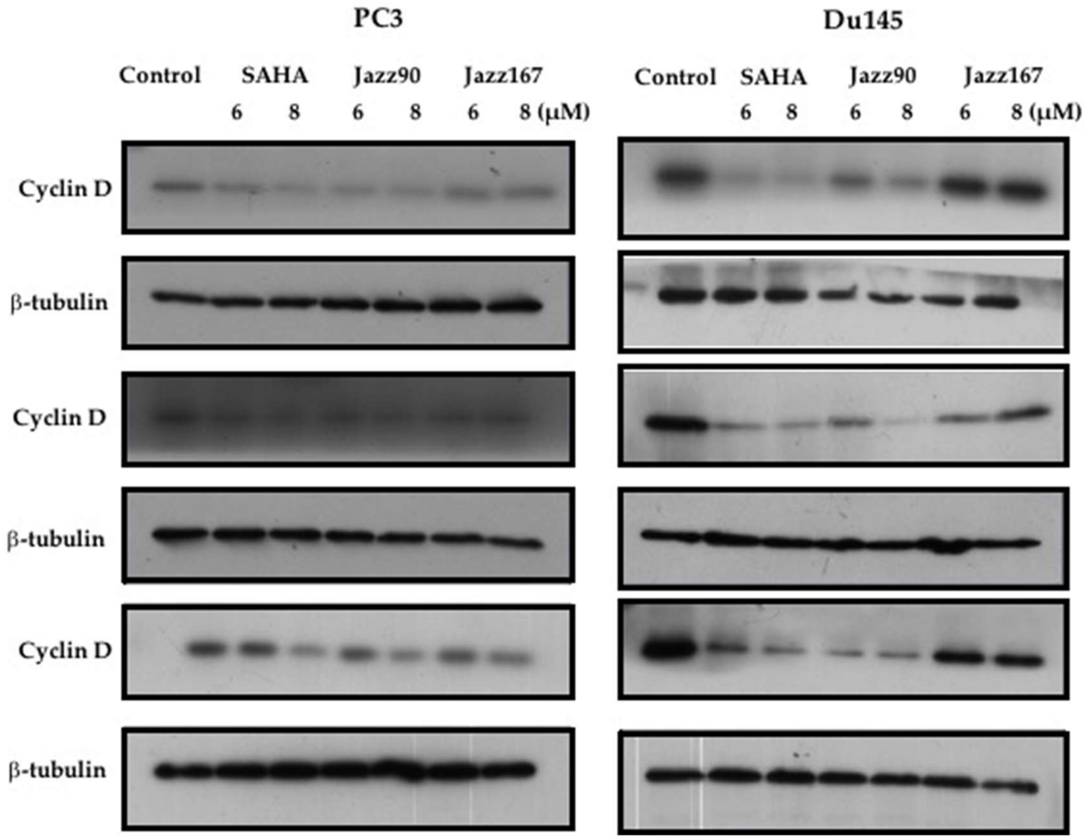

Figure S5- All blots for cyclin D in the cell lines PC3 and Du145. These blots were assessed via densitometry.

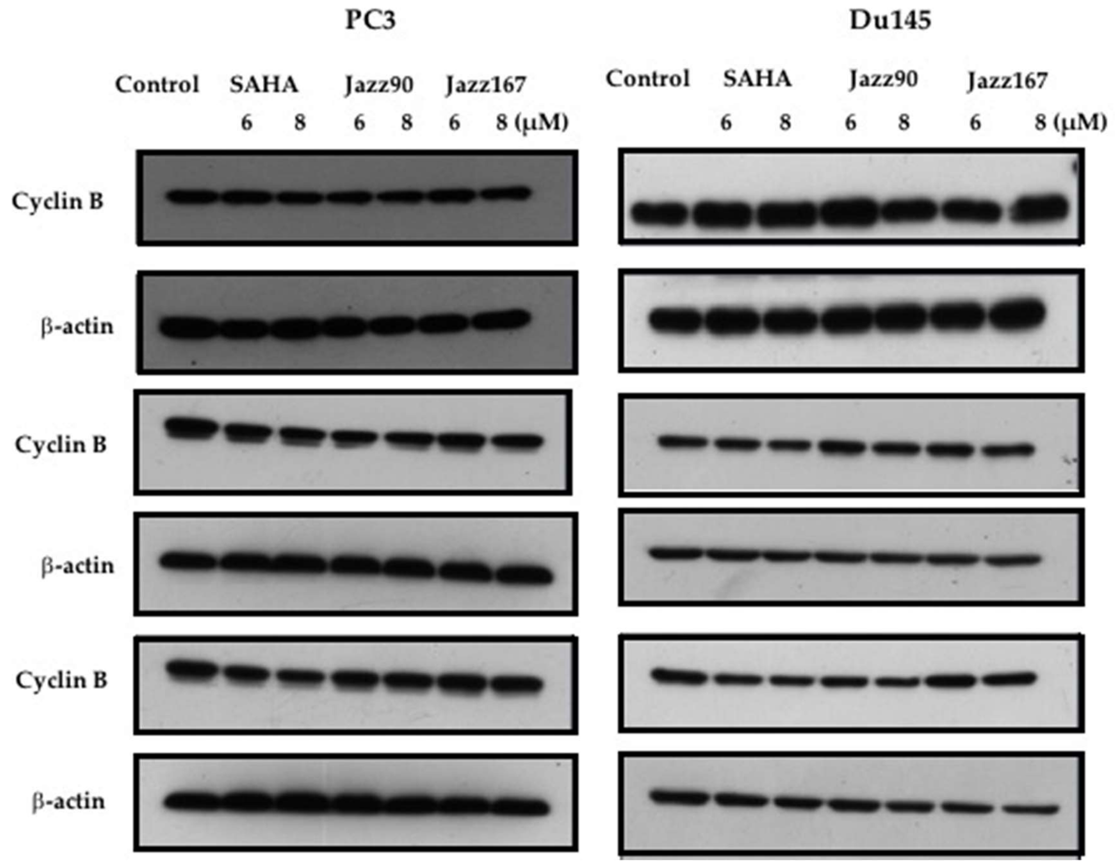

Figure S6- All blots for cyclin B in the cell lines PC3 and Du145. These blots were assessed via densitometry.

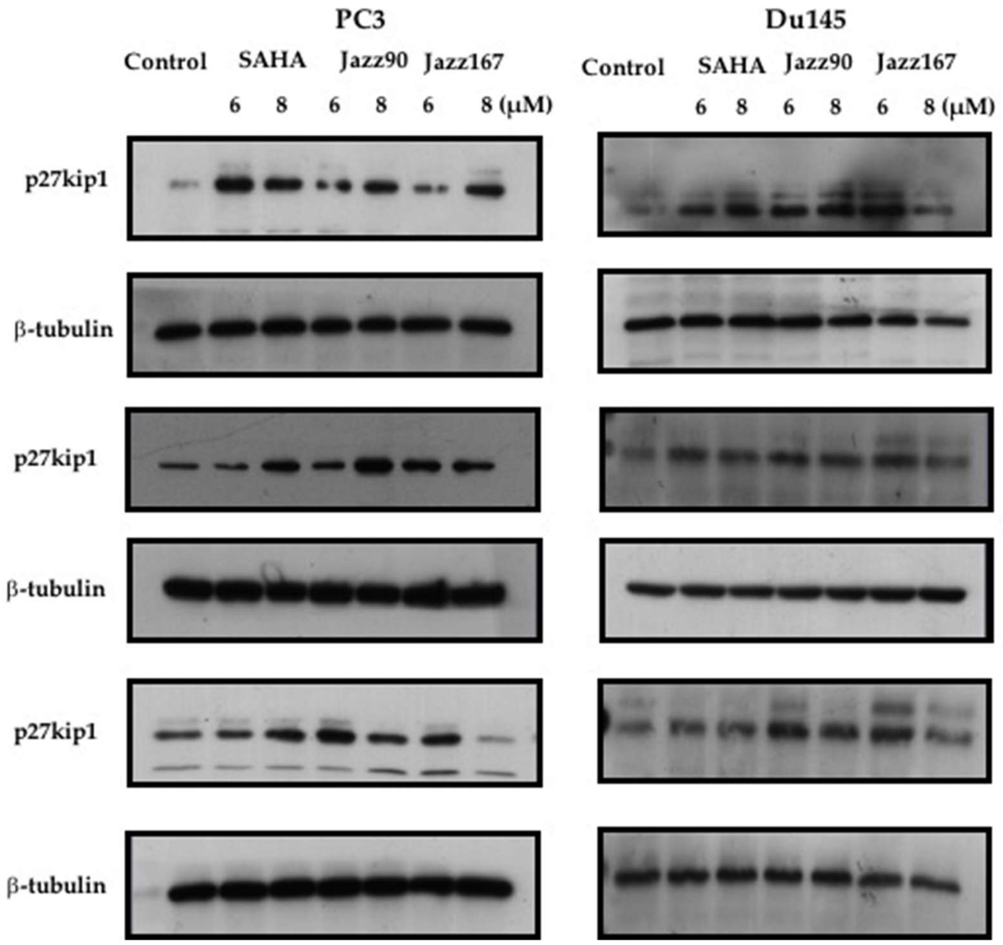

Figure S7- All blots for p27kip1 in the cell lines PC3 and Du145. These blots were assessed via densitometry.

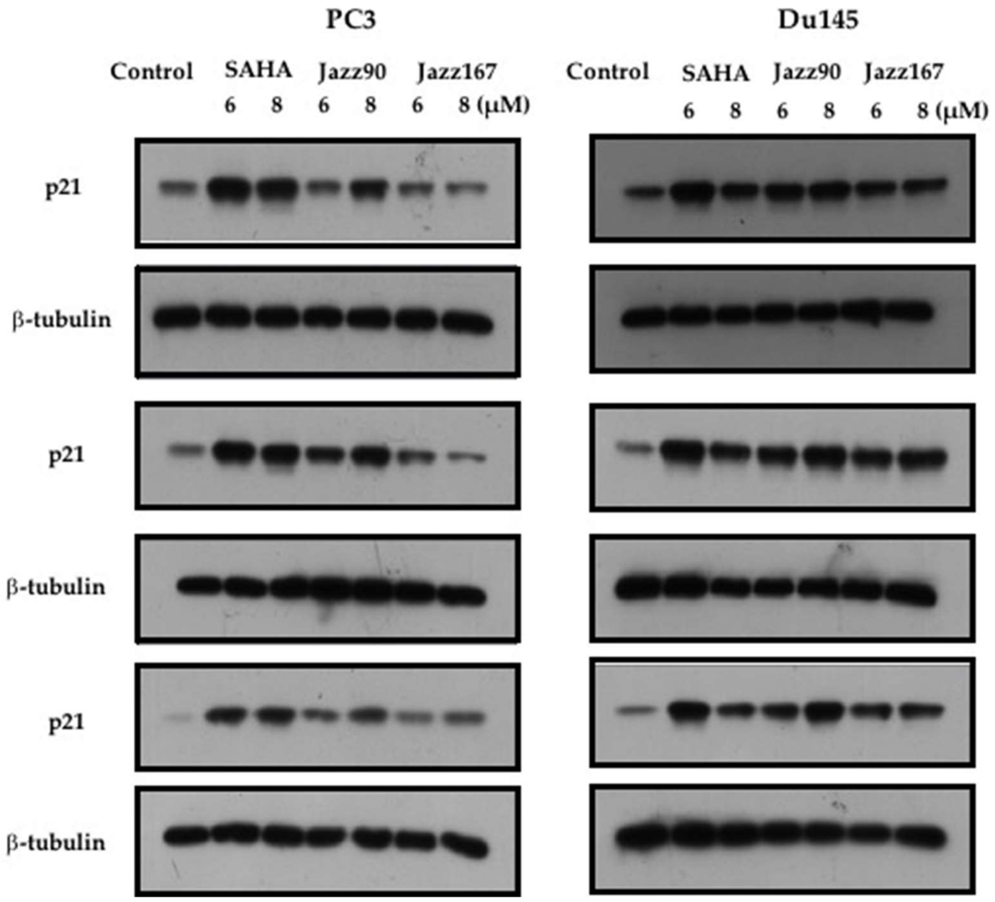

Figure S8- All blots for p21 in the cell lines PC3 and Du145. These blots were assessed via densitometry.

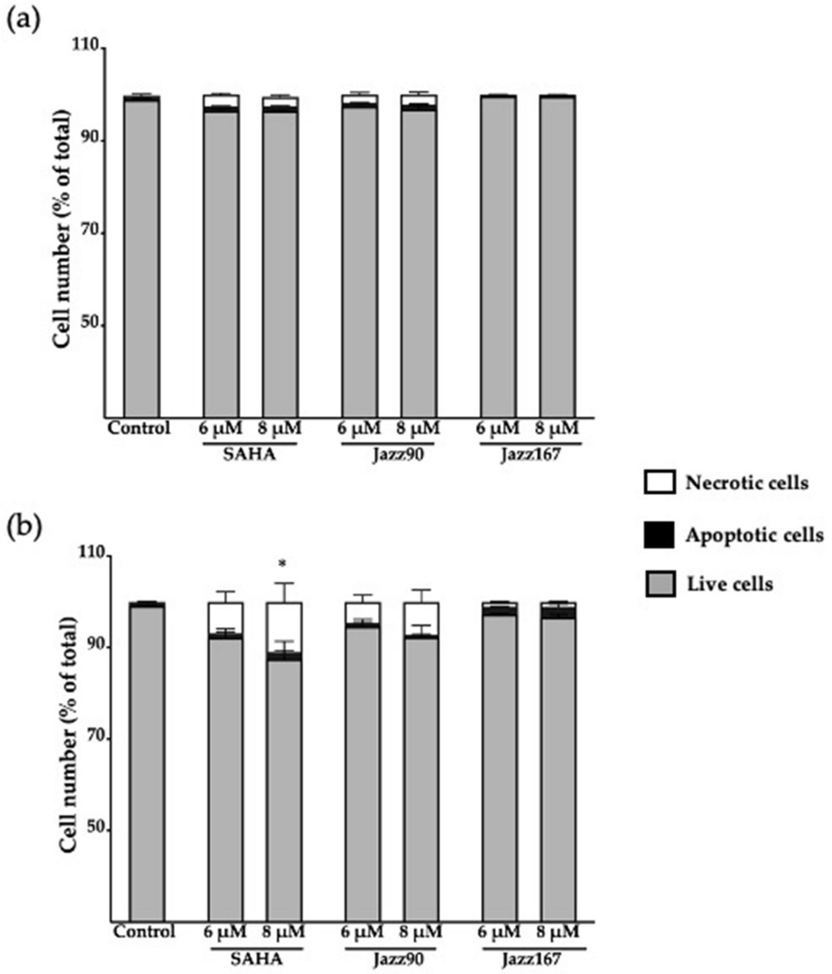

Figure S9. Analysis of apoptosis and necrosis in DU145 cells. Cells were treated with SAHA, Jazz90 and Jazz167 at concentrations of 6 and 8 μM. Vehicle control cells were treated with 0.5% DMSO. Cells were harvested at (a) 24 and (b) 48 hours, after which the cells were stained with Annexin V and PI. Stains were measured using flow cytometry. Bars represent the mean proportion of live, apoptotic and necrotic cells ± S.E.M. from 3 independent experiments. Data were analyzed using a two-way ANOVA coupled with a Bonferroni's post-hoc test. * indicates significant differences in necrosis relative to the control, $p < 0.05$.

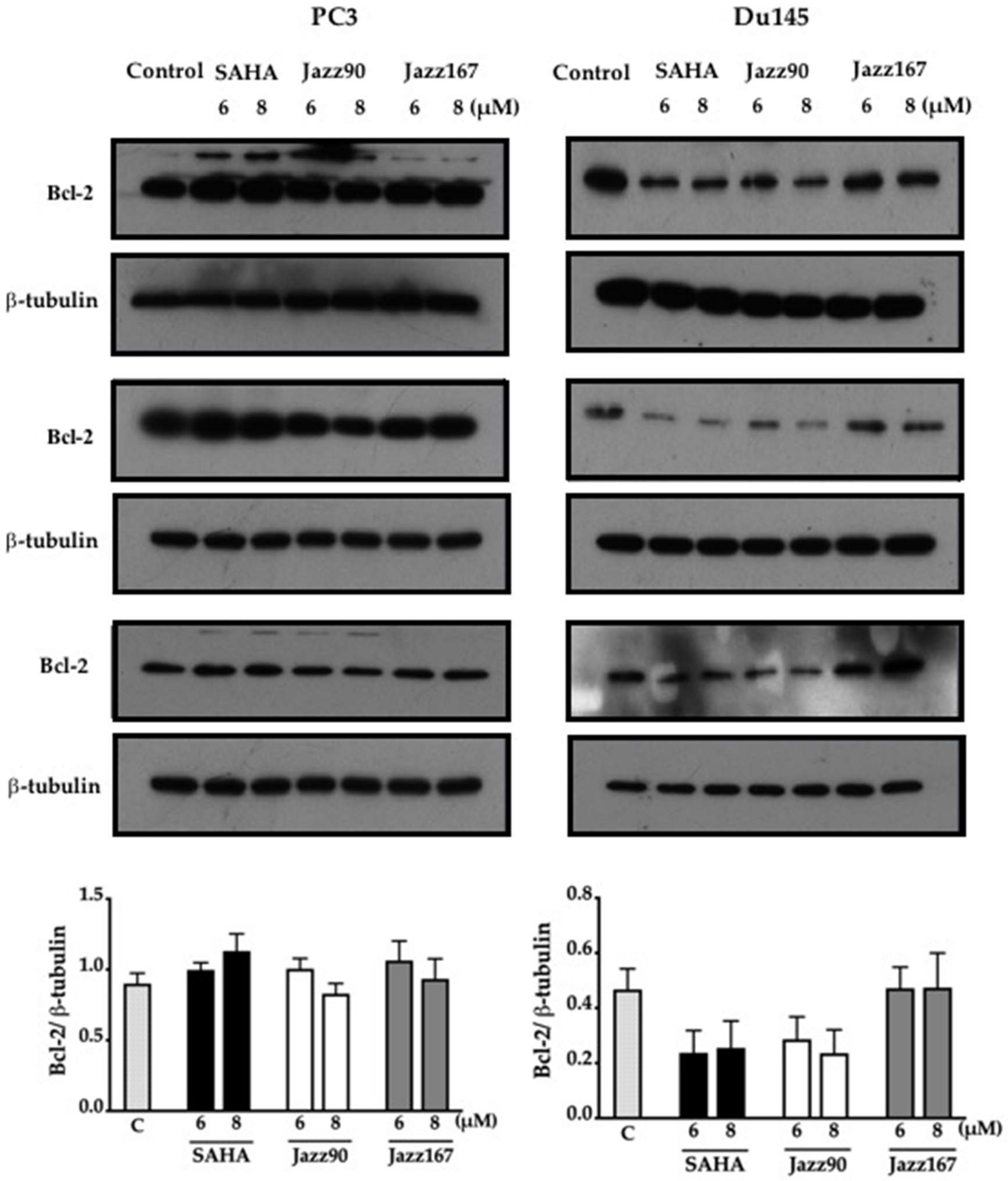

Figure S10- Expression profile of apoptotic protein Bcl-2 in PC3 and Du145 cells. No significant differences were found between the expression of Bcl-2 in drug treated cells compared to vehicle control. Mean (+/-) S.E.M was calculated from three independent experiments. One way ANOVA with Bonferroni's posthoc test was used to measure statistical differences. None were significantly different.

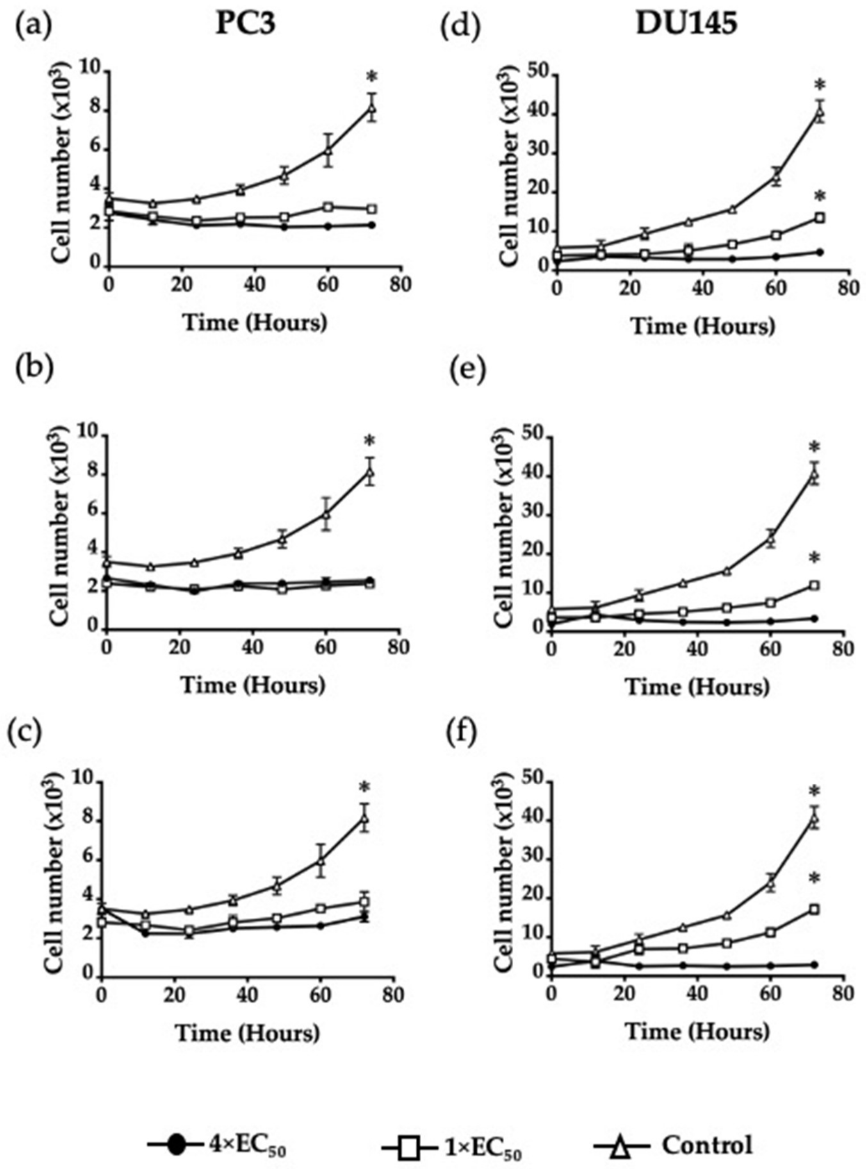

Figure S11. Compound withdrawal effects in prostate cancer cells. PC3 cells were treated with (a) SAHA, (b) Jazz90 and (c) Jazz167, and DU145 cells were treated with (d) SAHA, (e) Jazz90 and (f) Jazz167 at 1×EC_{50} and 4×EC_{50} concentrations for 72 hours. Control cells were treated with 0.5% DMSO. Compounds were removed and the cell numbers were measured for a period of 72 hours. Symbols represent cell numbers ± S.E.M from 3 independent experiments conducted in triplicate. Data were analyzed using a two-way ANOVA coupled with a Bonferroni's post-hoc test. * indicates significant differences between the cell number at withdrawal time zero and 72 hours, $p < 0.05$.
